# Effects of Combined Exposure to Lead and High-Fat Diet on Bone Quality in Juvenile Male Mice

**DOI:** 10.1289/ehp.1408581

**Published:** 2015-04-10

**Authors:** Eric E. Beier, Jason A. Inzana, Tzong-Jen Sheu, Lei Shu, J. Edward Puzas, Robert A. Mooney

**Affiliations:** 1 Center for Musculoskeletal Research,; 2 Department of Environmental Medicine, and; 3 Department of Pathology and Laboratory Medicine, University of Rochester Medical Center, Rochester, New York, USA

## Abstract

**Background:**

Lead (Pb) exposure and obesity are co-occurring risk factors for decreased bone mass in the young, particularly in low socioeconomic communities.

**Objectives:**

The goal of this study was to determine whether the comorbidities of Pb exposure and high-fat diet–induced obesity amplify skeletal deficits independently associated with each of these risk factors, and to explore associated mechanisms of the observed deficiencies.

**Methods:**

Five-week-old male C57BL/6J mice were placed on low-fat (10% kcal, LFD) or high-fat (60% kcal, HFD) diets for 12 weeks. Mice were exposed to lifetime Pb (50 ppm) through drinking water.

**Results:**

HFD was associated with increased body mass and glucose intolerance. Both HFD and Pb increased fasting glucose and serum leptin levels. Pb and HFD each reduced trabecular bone quality and together had a further detrimental effect on these bone parameters. Mechanical bone properties of strength were depressed in Pb-exposed bones, but HFD had no significant effect. Both Pb and HFD altered progenitor cell differentiation, promoting osteoclastogenesis and increasing adipogenesis while suppressing osteoblastogenesis. In support of this lineage shift being mediated through altered Wnt signaling, Pb and non-esterified fatty acids in MC3T3 cells increased *in vitro* PPAR-γ activity and inhibited β-catenin activity. Combining Pb and non-esterified fatty acids enhanced these effects.

**Conclusions:**

Pb and HFD produced selective deficits in bone accrual that were associated with alterations in progenitor cell activity that may involve reduced Wnt signaling. This study emphasizes the need to assess toxicants together with other risk factors relevant to human health and disease.

**Citation:**

Beier EE, Inzana JA, Sheu TJ, Shu L, Puzas JE, Mooney RA. 2015. Effects of combined exposure to lead and high-fat diet on bone quality in juvenile male mice. Environ Health Perspect 123:935–943; http://dx.doi.org/10.1289/ehp.1408581

## Introduction

Harmful effects of exposure to elevated lead (Pb) levels on human health have been well documented. In particular, the skeletal system is thought to be at higher susceptibility to these adverse effects because the Pb body burden (roughly 75% of exposure at any given time) is retained within the mineralized compartment of bone. Indeed, data from the Third National Health and Nutrition Examination Survey revealed that elevated Pb exposure is inversely correlated with femoral bone density and is associated with osteoporosis (Campbell and Auinger 2007; Nash et al. 2004). Rodent models have revealed a marked impairment of bone mineralization and density with Pb (Beier et al. 2013; Monir et al. 2010), predominantly in trabecular bone, where Pb accumulates (Farias et al. 2005).

The accumulation and influence of Pb in the skeleton do not occur in isolation but can coexist alongside numerous other risk factors that can also influence skeletal health, including host-related risk factors, genetic variations, or other environmental toxicant exposures. One such risk factor is obesity, which, like Pb exposure, can occur early in childhood and continue throughout life. Co-occurrence is especially frequent for low socioeconomic status populations. These communities have a higher propensity for childhood obesity (Fox et al. 2009; O’Dea and Wilson 2006), with a reported prevalence of 14.6% according to the Center for Disease Control and Prevention (CDC) Pediatric Nutrition Surveillance System (CDC 2009). This population also experiences disproportionately greater levels of environmental Pb exposure due to Pb persistence in old residences and its contamination of inner-city soils. The incidence of Pb exposure, as with obesity, appears to have lasting negative impact on skeletal growth and attainment of peak bone mass, and both are recognized as etiological factors in the development of osteoporotic-like bone disease (Ballew et al. 1999; Kafourou et al. 1997; Kawai and Rosen 2010; Pollock et al. 2011; Wosje et al. 2009).

Several aspects of the obesity phenotype appear to mediate its negative effect on bone health. The first is an increase in proadipogenic factors secreted by adipose tissue and bone. The hormone leptin is increased with obesity, and genetic models have shown that leptin can negatively influence bone mass indirectly via serotonin suppression in the central nervous system (Karsenty and Oury 2010). Adipokines have also been reported to directly suppress osteoblast differentiation (Kawai and Rosen 2010). Altered Wnt signaling in obesity may have implications in bone. Chen et al. (2010b) reported that rats on a high-fat diet (HFD) had deficiencies in bone mass gain that were associated with significant depression of Wnt/β-catenin signaling. Similar to HFD, Pb has been implicated in the depression of Wnt signaling that is linked to a reduction in bone mass (Beier et al. 2013).

The similarity of deficits in bone density and other skeletal pathologies associated with Pb and obesity suggest that they may share one or more biological mechanisms. Pluripotent mesenchymal stem cells (MSCs) give rise to osteogenic and adipogenic cells that exist in a reciprocal relationship based on culture condition *in vitro* or local microenvironment *in vivo* (Krishnan et al. 2006). MSCs express low, biologically relevant levels of both proadipogenic transcription factors PPAR-γ and C/EBPα and pro-osteoblast transcription factors such as Runx2 and osterix. Negative feedback between both classes of transcription factors maintains the cells in an undifferentiated state, whereas an imbalance leads to differentiation (Takada et al. 2009). Certain Wnt molecules and downstream signaling events are known to regulate the balance between these factors, and thus are described as gatekeepers for the bifurcation of MSCs to various cell fates (Ross et al. 2000).

In this investigation, we provide evidence that both Pb and HFD depress the Wnt signaling pathway, thus possibly altering MSC fate through this important pathway. These effects may explain our observations that Pb exposure and HFD-induced obesity alone, and more effectively together, lead to decreased bone mass and quality.

## Materials and Methods

*Animal Pb exposure and treatment with HFD*. Animal studies were performed in accordance with protocols approved by the University of Rochester’s Committee on Animal Resources that maximize humane treatment and alleviation of suffering. C57BL/6J dams received either 0 or 50 ppm Pb acetate–treated water, beginning at delivery of litters. Optimization of dosage and the treatment protocol have been previously described (Carmouche et al. 2005). After weaning, male offspring were continued on the same water treatments. At 5 weeks of age, pups from each group were placed either on an *ad libitum* low-fat diet (LFD) or HFD (Open Source Diets; Research Diets Inc.) (Reeves et al. 1993). Males from multiple litters were randomized to avoid placing littermates in the same experimental groups. Low-fat feed (D12450B) was formulated with 10% kcal from fat, 70% carbohydrate (35% sucrose content), and 20% protein (with casein as the sole protein source). High-fat feed (D12492) was formulated from 60% kcal from fat, 20% carbohydrate (7% sucrose content), and 20% protein. The 10% kcal from fat provided by the LFD is very similar to the standard mouse chow used at the University of Rochester (LabDiet 5010), which contains 12.7% kcal from fat. At 3, 6, or 12 weeks on diets, mice (*n* = 5–6) were harvested for bone and metabolic phenotyping. Blood Pb levels were determined by anodic stripping voltammetry using the Lead Care II system (Magellan Diagnostics), and bone Pb was determined by atomic absorption on the proximal half of tibias (*n* = 4). *In vivo* body fat composition of trunk, legs, and arms of mice was measured by dual-energy X-ray absorptiometry (DXA) (Lunar Prodigy Advance; GE Healthcare). Glucose tolerance tests were performed at 11 weeks on diet as previously described (Brown et al. 2014) following a 24-hr fasting period (*n* = 5). For additional details, see Supplemental Material, “Animals” and Figure S1.

*Serum bone remodeling markers, leptin, and Wnt signaling*. Serum levels of the osteoblastic marker type 1 procollagen (P1NP)and the osteoclast marker TRACP5b were measured by an ELISA according to the manufacturer’s directions (Nordic Biosciences Diagnostic). Serum levels of leptin and sclerostin (ALPCO Diagnostics), DKK1 (R&D Systems), and total non-esterified fatty acids (NEFA) (Wako Diagnostics) were also measured using an ELISA method.

*MicroCT and biomechanics*. Mouse femurs and tibiae were imaged by micro computed tomography (microCT) (VivaCT40; Scanco Medical) using an integration time of 300 msec, energy of 55 kVp, and intensity of 145 μA. Images were reconstructed to an isotropic voxel size of 10.3 μm. Analyses of trabecular and cortical bone were performed as previously described (Inzana et al. 2013). For trabecular analysis in the distal femoral metaphysis, a 200-μm region proximal to the growth plate was used for quantification. The trabecular bone morphology of the femoral metaphysis, including the bone volume to total tissue volume ratio (BV/TV), connective density (Conn.D), trabecular number (Tb.N), trabecular thickness (Tb.Th), trabecular spacing (Tb.Sp), and structural model index (SMI), were determined using Scanco’s three-dimensional (3D) analysis tools (direct model). Femoral cortical bone was measured at the mid-diaphysis by averaging over a 200-μm region (19 slices). 3D rendering allowed generation of cortical thickness data. A sliding caliper was used to measure femur diameter at the mid-diaphysis.

Six femoral specimens were analyzed per group for three-point flexural testing with the anterior surface in tension using an 8-mm support span at a displacement rate of 3 mm/min (Instron 4465/5500; Instron Corp.). Bones were set with a 1.5 N preload and monitored until failure using an Instron 8841 DynaMight^™^ Axial Testing System (Instron Corp.) with a 50 N load cell. Stress–strain data were estimated from the measured load-displacement curves using microCT measurements of the moment of inertia and maximum radius to the mediolateral axis. The bending load data were plotted against displacement data, which were normalized by the polar moment of inertia of each specimen (apparent strain), to determine the maximum strength (max force), maximum stress (max stress), tensile stiffness (stiffness), maximum toughness, bending modulus (modulus), and post-yield strain. The yield point was determined by a 0.2% strain offset. Apparent stresses were estimated by normalizing the loads by the total cross-sectional bone area of each femur.

*Bone histology*. After harvesting, leg bones were fixed in 10% formalin for 3 days, decalcified for 2 weeks in 14% EDTA, processed, and embedded in paraffin. Medial sections were stained with Alcian blue hematoxylin and orange G (ABH) for histologic and histomorphometric analysis. Histomorphometric analysis was performed using OsteoMeasure analysis software (OsteoMetrics) according to the manufacturer’s methods, and using published nomenclature and units (Dempster et al. 2013). The region for tibial trabecular bone analysis was a 1.23-mm^2^ area below the growth plate. For intramedullary fat analysis, the number and size of fat vacuoles were quantified.

*Osteoclast, osteoblast, and adipocyte formation assays*. MSCs were harvested from bone marrow of femurs according to published methods (Zhang et al. 2002). MSCs were divided for differentiation assays.

Osteoclast formation assay. Cells from LFD and HFD mice were seeded (5 × 10^4^/well) with and without Pb in 96-well plates and cultured for 5–7 days in 10% fetal bovine serum (FBS) α-MEM (minimum essential medium) containing conditioned medium (1:50) from an M-CSF (macrophage colony-stimulating factor)–producing cell line and RANKL (receptor activator of nuclear factor kappa-B ligand; 10 ng/mL; R&D Systems) as described previously (Yamashita et al. 2007). Cells were then stained for TRAP (tartrate-resistant acid phosphatase) activity to identify osteoclasts. TRAP-positive osteoclast area was determined by histomorphometry.

Osteoblast formation. MSCs were seeded in 12-well plates and cultured for 21 days in osteogenic α-MEM as described previously (Ryan et al. 2007). Cultures were then stained with alizarin red to assess matrix mineralization.

Adipocyte formation. Cells were seeded in 12-well plates and cultured for 10 days in adipogenic DMEM (Dulbecco’s Modified Eagle medium) as described previously (Beier et al. 2013). Cultures were stained with Oil Red O and quantified by dissolving stain in 4% IGEPAL (Sigma) and measuring absorption at 490 nm.

*Quantitative real-time polymerase chain reaction (qPCR) and luciferase assays*. MC3T3-E1 cells, acquired from ATCC, were cultured in 10% FBS α-MEM containing 50 μg/mL ascorbate. NEFA (the fatty acids oleate and palmitate, 1:2 mixture; Sigma) was dissolved in 95% ethanol at 60°C and mixed with bovine serum albumin (10%), which yielded a stock concentration of 5 mM. Pb acetate was made to 3 mM in distilled H_2_O. After a 24-hr treatment, total RNA was isolated using QIAGEN mini columns and reverse transcribed using the iScript cDNA synthesis kit (Bio-Rad). qPCR reactions were carried out using PerfeCTa SYBER green (Quanta Biosciences) according to manufacturer’s protocols. The genes of interest were normalized to β-actin expression.

Transfections and luciferase activity assays were performed as described previously (Zuscik et al. 2007). In brief, MC3T3 cells were transfected with reporters for PPAR-γ (PPRE-Luc), Wnt/β-catenin signaling (TOPFLASH), and 7-kb human sclerostin promoter (SOST-Luc) (Yu et al. 2011). Transfections were performed using Superfect (QIAGEN). The SV40 Renilla-Luc plasmid was cotransfected to facilitate determination of transfection efficiency. The DNA:transfection-reagent ratio was 1:3 (weight/volume) with 2 μg reporter of interest and 10 ng of SV40 Renilla-luc. Within 12 hr, cells were exposed to various treatments; 48 hr later, cells were lysed and extracts were prepared using the Dual Luciferase Assay System (Promega). An Optocomp luminometer (MGM Instruments) was used to measure luminescence in the extracts. Treatments of transfected cells for 48 hr were as follows: 100 ng/mL Wnt3a, alone or in combination with 1 μM or 5 μM Pb; and 400 μM NEFA or Pb plus NEFA.

*Statistics*. Data are presented as mean ± SEM. The overall differences between treatment groups were compared using two-way analysis of variance (ANOVA) between groups to examine the effects of diet, Pb exposure, and their interaction (Pb × diet). When significant interactions occurred, we applied Bonferroni’s correction to evaluate whether the differences due to diet were significant within each Pb treatment after accounting for multiple comparisons. Multiplicity-adjusted *p*-values < 0.05 were considered to be statistically significant. One-way ANOVA with Bonferroni’s multiple comparison test was used to compare means. GraphPad Prism, Version 6 (GraphPad Software) was used for all statistical analyses.

## Results

**Figure 1:**
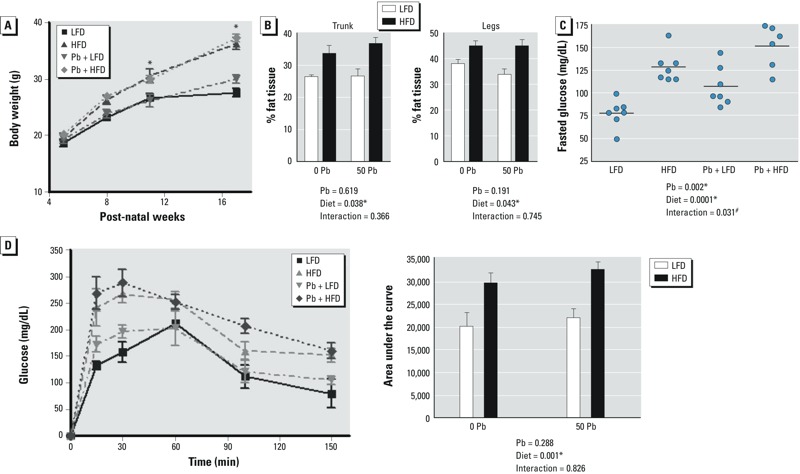
Effect of dietary fat and Pb (50 ppm) on body weight and glucose in male mice placed on HFD or LFD for 12 weeks. (*A*) Weight gain of mice recorded over the course of the experiment. (*B*) Body fat composition in the trunk and legs of mice at 12 weeks by DXA scans. (*C*) Fasting glucose levels analyzed at the start of the glucose tolerance test. (*D*) Blood glucose levels measured over time after an intraperitoneal injection of glucose (left); area under the curve analysis shows significant differences between LFD and HFD (right). Data are mean ± SEM of 5 mice/group. *p < 0.05 for effect of Pb or diet. ^#^*p* < 0.05 for interaction of Pb and diet.

*Effects of HFD and Pb exposure on glucose levels in growing male mice*. To investigate the mechanism of Pb- and obesity-induced bone loss, we exposed male C57BL/6J mice to normal or Pb-treated drinking water beginning at birth. The average blood Pb level in treated mice was 8 μg/dL at 5 weeks of age and 4 μg/dL at 17 weeks of age. Diet had no effect on blood Pb levels. Note that a 5-μg/dL blood Pb level places a child in the top 2.5% of tested children (CDC 2012). When mice were 5 weeks of age [equivalent to 10-year-old children (Grounds et al. 2008)], they were placed on an LFD or HFD. Mice on the HFD gained more weight than LFD mice within 6 weeks, but Pb exposure had no effect on weight (Figure 1A). Fat composition was significantly higher in the trunk and legs of HFD mice compared with controls (Figure 1B). Pb exposure had no effect on fat composition.

After 11 weeks on diet, fasting glucose levels were elevated in the HFD (1.68 times higher) and Pb (1.45 times higher) groups, with a further increase in mice receiving the combination of HFD plus Pb (1.99 times higher), compared with LFD controls (Figure 1C). Glucose tolerance tests showed impaired glucose handling in both HFD alone and HFD plus Pb groups (Figure 1C,D). No change was observed with Pb alone, and Pb did not alter the magnitude of the HFD effect.

**Figure 2:**
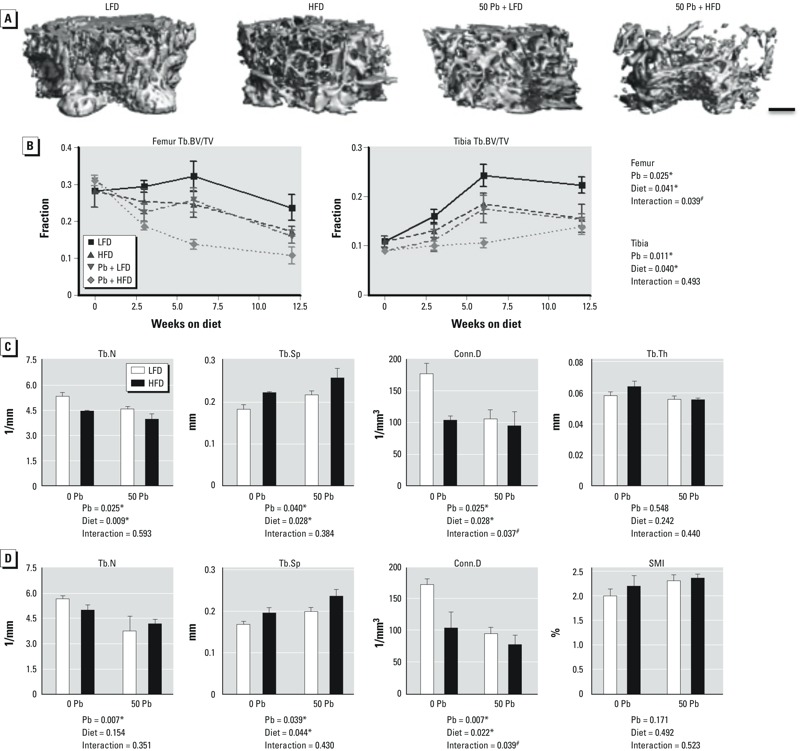
Effect of dietary fat and Pb (50 ppm) on bone mass and quality in male mice placed on HFD or LFD for 12 weeks. (*A*) 3D microCT images of representative transverse sections of the distal femurs from each diet and Pb group at 12 weeks on diet (bar = 250 μm). (*B*) Quantitative microCT determination of Tb.BV/TV over time in distal femurs and proximal tibias as a function of treatment; statistics are shown for mice at 12 weeks on diet. Trabecular bone from the femur (*C*) and tibia (*D*) were further analyzed at 12 weeks for additional bone parameters. Abbreviations: Conn.D, connective density; Tb.BV/TV, trabecular bone volume/total tissue volume; Tb.Th, trabecular thickness; Tb.N, trabecular number; Tb.Sp, trabecular spacing; SMI, structural model index. Data are mean ± SEM of 5 mice/group. *p < 0.05 for effect of Pb or diet. ^#^*p* < 0.05 for interaction of Pb and diet.

*Combined effects of Pb exposure and HFD on bone mass*. Pb-exposed mice that consumed an HFD had significantly increased tibial bone Pb deposition at 6 weeks (2.4 times higher) and 12 weeks (2.0 times higher) on diet compared with LFD Pb mice (Table 1). These levels of bone Pb are comparable to tibial Pb levels of 10–20 ng/mg (or ppm) attained by at-risk children and youths (Needleman et al. 2002; Rosen et al. 1993). Although no gross differences in water ingestion were observed between diet groups, ingestion was not quantitated. Thus, it cannot be ruled out that increased water consumption by the HFD group, and therefore greater exposure to Pb, could be a contributing factor to the greater accumulation of Pb in the bones of HFD mice. To examine the impact of Pb deposition and HFD on the skeleton, we analyzed trabecular bone in the femur and tibia by microCT. Before the LFD and HFD were initiated (5 weeks of age), no significant differences were observed between the Pb-exposed mice and controls (*n* = 5). Reduced bone volume at 12 weeks was found in femurs of mice fed HFD (–27.0%) or treated with Pb (–32.9%) compared with LFD mice (Figure 2A,B). In mice receiving HFD plus Pb, the decrement in BV/TV was even greater (–46.3%). Significant BV/TV differences were also observed in the tibia of the Pb and HFD groups, with differences even larger in the Pb plus HFD group. Additional bone parameters in the femur were significantly changed by Pb or HFD (Tb.N, Tb.Sp, and Conn.D) compared with the LFD control (Figure 2C), with a significant interaction of Pb × HFD only with respect to Conn.D. No alteration was seen in Tb.Th. Phenotypic alterations were also observed in the trabecular bone of the tibia by Pb or HFD, with a significant interaction of Pb × HFD only with respect to Conn.D (Figure 2B,D). No significant change in trabecular convexity (structural model index, SMI) was observed. These changes were consistent with what has been previously shown with Pb alone (Beier et al. 2013) and HFD alone (Inzana et al. 2013). No change was observed in cortical thickness or diameter at any time point (data not shown). Therefore, the major effect of Pb and HFD during this period of bone mass accrual was a decrease in trabecular bone.

**Table 1:** Bone Pb levels in mice (ng Pb/mg dry wt of tibial bone) at 6 and 12 weeks on diet.

Time point	LFD	HFD	50 ppm Pb + LFD	50 ppm Pb + HFD
6 weeks	0.30 ± 0.01	0.24 ± 0.04	9.95 ± 1.02^*a*^	23.46 ± 2.47^*a*^^,^^*b*^
12 weeks	0.14 ± 0.03	0.10 ± 0.01	7.27 ± 0.47^*a*^	14.30 ± 3.05^*a*^^,^^*b*^
Data are mean ± SEM. Pb was determined in the proximal half of mineralized tibiae by atomic absorption (*n *= 4/group). ^***a***^*p* < 0.05 for effect of 50 ppm Pb exposure by two-way ANOVA. ^***b***^Multiplicity-adjusted *p* < 0.05 for interaction effect (50 ppm Pb + LFD vs. 50 ppm Pb + HFD) by two-way ANOVA with Bonferonni’s correction for multiple comparisons.				

*Effects of Pb and HFD on bone strength*. Pb but not HFD had a statistically significant impact on the flexural strength of femurs from mice after a 12-week diet treatment (Table 2). Pb alone significantly decreased stiffness and max force compared with controls, suggesting a deficit in bone mineral properties. HFD alone was associated with decreases in the same parameters that trended toward significance. No additional changes were observed with combined Pb and HFD treatments. After adjusting the bending force by the cross-sectional bone areas to estimate the apparent stresses, there were no alterations in bone elasticity, mechanical stress, or toughness in either treatment group. However, post-yield strain was significantly reduced as a consequence of Pb exposure, which indicates a significant reduction in the inductility of the bone.

**Table 2:** Three-point flexural properties of femurs in Pb-treated and diet-fed mice after 12 weeks on diet.

Treatment	Stiffness (*n*/mm)	Modulus (MPa)	Max force (*n*)	Max stress (MPa)	Toughness (mJ/mm^3^)	Post-yield strain (%)
LFD	75.5 ± 4.7	4,293 ± 488	16.9 ± 0.8	121 ± 8	4.49 ± 0.29	3.30 ± 0.13
HFD	70.7 ± 5.1	3,902 ± 558	15.2 ± 1.2	128 ± 16	4.62 ± 0.55	3.28 ± 0.18
50 ppm Pb + LFD	52.9 ± 6.1^*a*^	3,981 ± 603	14.6 ± 0.6^*a*^	131 ± 8	4.66 ± 0.50	2.68 ± 0.13^*a*^
50 ppm Pb + HFD	66.6 ± 2.3^*a*^	3,989 ± 146	14.9 ± 0.7^*a*^	117 ± 8	3.61 ± 0.33	2.26 ± 0.25^*a*^
Data are mean ± SEM (*n *= 6/group). ^***a***^*p* < 0.05 for effect of Pb exposure by two-way ANOVA.						

**Figure 3:**
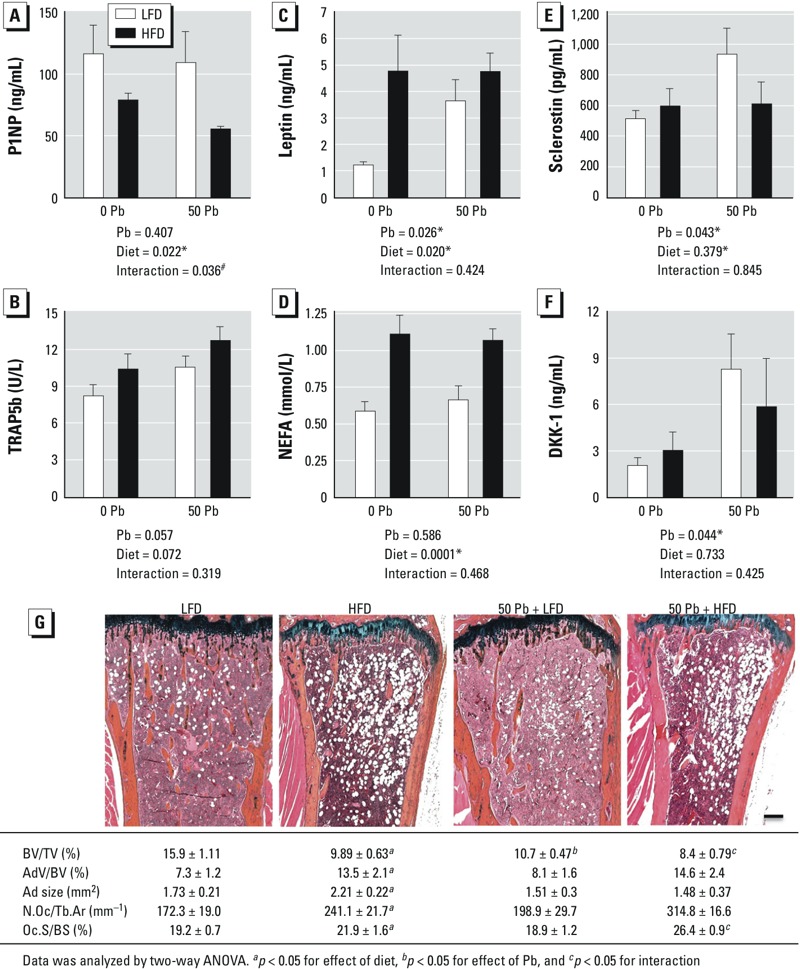
Effects of HFD and Pb (50 ppm) on histologic and serum bone parameters; Pb-exposed and control mice were placed on LFD or HFD for 6 weeks. Serum bone formation marker P1NP (*A*), resorption marker TRAP5b (*B*), leptin (*C*), NEFA (*D*), sclerostin (*E*), and DKK1 (*F*) were measured using ELISA methods. Data are mean ± SEM of 5 mice/group. (*G*) Trabecular bone in the proximal tibia was assessed histologically by Alcian blue hematoxylin/orange G staining (bar = 100 μm); images are representative sections from treatment groups, selected for approximation to the median BV/TV of its group. Abbreviations: AdV/BV, adipocyte volume/bone volume; Ad size, adipocyte size; BV/TV, bone volume/ total tissue volume; N.Oc/Tb.Ar, number of osteoclasts/trabecular bone area; Oc.S/BS, osteoclast surface/bone surface. The effects of Pb and/or HFD on osteoclast and adipogenic parameters were calculated and are presented at the bottom of each image. Data represent mean ± SEM for 3 mice/group. *p < 0.05 for effect of Pb or diet. ^#^*p *< 0.05 for interaction of Pb and diet.

*Altered bone histomorphometry and serum markers in Pb- and HFD-treated mice*. The bone formation marker P1NP was significantly decreased in the HFD group and was further decreased in combination with Pb compared with controls after 6 weeks on diets (Figure 3A). TRAP5b, an indicator of osteoclast number (N.Oc), was elevated in both Pb and HFD groups, but not significantly so. The increase was greater when combined, but the interaction was not significant (Figure 3B). Leptin levels were significantly elevated by both HFD and Pb, but these treatments did not have any interaction (Figure 3C). NEFA were elevated as a consequence of HFD, but Pb had no effect (Figure 3D). The Wnt inhibitors sclerostin (Figure 3E) and DKK1 (Figure 3F) were induced in Pb-exposed mice compared with controls, but HFD antagonized this effect although not significantly.

Histomorphometric analysis of bone in the proximal tibia after 6 weeks showed both Pb and HFD to be associated with a significant decrease in trabecular bone volume compared with LFD mice (Figure 3G) and significantly interacted to lower BV/TV. Only HFD increased bone marrow adiposity [adipocyte volume to tissue volume (AdV/TV), size]. In the presence of HFD, osteoclast parameters were significantly elevated [N.Oc and osteoclast surface (Oc.S)]. There was also a significant interaction between Pb and HFD in Oc.S but not N.Oc. These results, together with the biomarker data, suggest that the enhanced loss of bone mass could in part be due to an increase in bone resorption.

**Figure 4:**
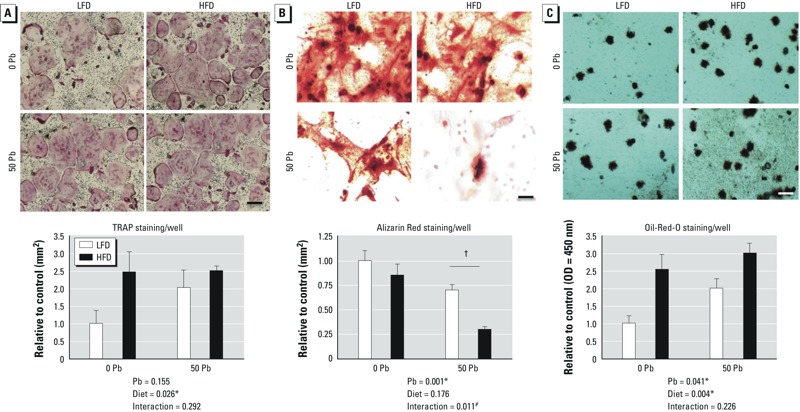
Effects of Pb (50 ppm) and HFD treatment on osteoclast and adipocyte formation, and on osteoblastic bone formation potential. Bone marrow stromal cells (MSCs) were isolated from femoral bones after 6 weeks on diet. (*A*) Osteoclast formation was evaluated by positive TRAP expression in MSCs after treatment with M-CSF and RANKL for 7 days. (*B*) Alizarin Red staining after 21 days in osteogenic media determined osteoblastic bone mineralization. (*C*) Adipocyte formation was measured by Oil Red O staining after 10 days in adipogenic media. Representative stains of cell cultures from each group (top; bar = 100 μm) were quantified (bottom); data are mean ± SEM for four trials. *p < 0.05 for effect of Pb or diet. ^#^*p* < 0.05 for interaction of Pb and diet. ^†^*p* < 0.001 for post hoc comparison of Pb-LFD vs. Pb-HFD.

*Effects of Pb and HFD on osteoclastic and adipogenic potential, and on osteogenic formation*. Bone marrow cell aspirates from HFD-treated mice gave rise to more TRAP-positive osteoclasts than those from LFD controls (Figure 4A). Pb appeared to have a similar, although not significant, effect. The osteogenic capacity of the cells, as assessed by alizarin red staining of *in vitro* mineralization, was decreased in Pb-exposed mice but was not significantly changed with HFD (Figure 4B). However, Pb plus HFD showed a significant interaction to further decrease the number of mineralized clusters. Adipogenic formation was significantly enhanced by either Pb or HFD (Figure 4C). These results suggest a fate switch in progenitor cells that promotes bone resorption and adipogenic formation at the expense of bone formation capacity (i.e., osteoblastogenesis).

**Figure 5:**
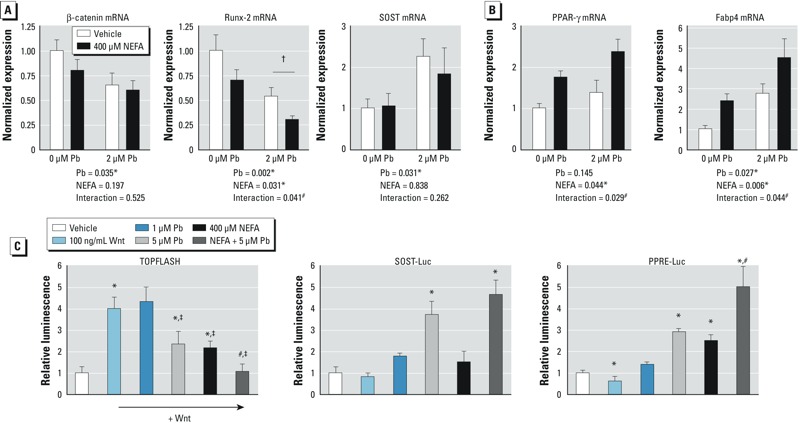
Combined effects of Pb and NEFA on osteogenic signaling and adipogenic signaling in MC3T3 cells. Cells were exposed to Pb, NEFA, or the combination of both for 5 days in α-MEM plus ascorbate. Expression profiles of osteoblastic genes (*A*) and adipogenic genes (*B*) were assessed by qRT-PCR. (*C*) Activity of luciferase reporter constructs for Wnt (TOPFLASH) was analyzed in response to 100 ng/mL Wnt 3a alone or Wnt 3a plus Pb (1 μM or 5 μM), NEFA (400 μM), or combined exposure. Sclerostin (SOST-Luc) and PPAR‑γ (PPRE-Luc) activities were tested in each exposure group. Data are mean ± SEM for three experiments. *p < 0.05 for effect of Pb, NEFA, or Wnt3a. ^‡^*p* < 0.05 vs. Wnt3a alone. ^#^*p* < 0.05 for interaction of Pb and NEFA. ^†^*p* < 0.005 for post hoc comparison of Pb-Veh and Pb-NEFA.

*Combined effect of Pb and HFD on Wnt and PPAR signaling*. Expression of the transducing molecule β-catenin (*Ctnnb1*) and the osteoblast differentiation transcription factor *Runx2* in MC3T3 cells was significantly decreased with exposure to 2 μM (41 μg/dL) Pb (Figure 5A), whereas NEFA reduced *Runx2* expression. The combination of Pb and NEFA further decreased expression of *Runx2* compared with controls. Sclerostin (*Sost*) expression was also elevated by Pb, but NEFA had no effect. Concurrently, mRNA levels of proadipogenic genes *Pparg* (PPAR-γ) and *Fabp4* (adipocyte protein 2) were elevated by NEFA, but Pb increased only *Fabp4* (Figure 5B). Maximum expression of the proadipogenic genes was attained with exposure to Pb plus NEFA. Note that chelation therapy is recommended for children with Pb levels of ≥ 45 μg/dL (CDC 2012). Thus, the levels used here are in the relevant range for children with serious lead exposure.

TOPFLASH reporter activity was significantly increased in response to Wnt3a. Interestingly, 5 μM Pb (104 μg/dL) or 400 μM NEFA blunted the Wnt3a response (Figure 5C). The combination of Pb plus NEFA extinguished activated Wnt signaling. Pb and NEFA had no effect on TOPFLASH activity under basal conditions. The SOST promoter activity was responsive to 5 μM Pb, but NEFA had little effect (Figure 5C). In contrast, PPAR-γ signaling was comparably increased by 400 μM NEFA and 5 μM Pb, with an additional effect when tested in combination (Figure 5C).

## Discussion

In environmental exposures, agents rarely exist in isolation but rather occur in the context of numerous other risk modifiers. Most epidemiological studies and animal models of Pb toxicity have examined the effects of Pb as an independent variable, not with other codependent variables. In this study we present evidence for enhanced skeletal deficits in young mice subjected to lifetime Pb exposure combined with HFD-induced obesity. Specifically, discernible decreases in trabecular bone mass and osteoblastic function were observed in response to Pb or HFD. The combination of Pb and HFD exacerbated these effects. Although a limitation of this study is the absence of dynamic measurements such as bone formation and mineral apposition rates of long bones that would have added to our understanding of the decreased bone mass, our data show that bones of mice exposed to Pb were more brittle and less resistant to biomechanical forces. Pb and NEFA elevated *in vitro* formation of adipogenic cells. These results suggest that, in part through deficits in osteoblast differentiation and reduction in Wnt signaling, these toxicants target progenitor MSCs and alter their ability to differentiate into appropriate cell lineages.

Differences in the potential mechanisms by which Pb and HFD inhibit Wnt signaling and affect related outcomes are apparent. Pb exposure produced robust serum elevations of the Wnt inhibitors sclerostin and DKK1. These inhibitors have been shown to be elevated in several bone pathologies including osteoporosis, immunization-induced bone loss, and multiple myeloma (Colucci et al. 2011; Gaudio et al. 2010; Pinzone et al. 2009). In addition, therapies targeting these molecules in preclinical studies have profoundly improved bone loss by promoting anabolic effects (Christoulas et al. 2009; Li et al. 2009). Sclerostin is a major product of osteocytes. We found low Pb-responsive expression of its gene, *Sost*, in MC3T3-E1 osteoblastic cells relative to Runx2 and β-catenin expression (based on normalized CT values). Although both sclerostin serum levels and its gene expression in osteoblastic MC3T3 cells doubled with Pb exposure, the serum changes are more likely arising from effects of Pb on osteocytes. Relative protein expression may differ, but our data suggest that inhibition of Wnt signaling by sclerostin is not the primary mechanism for the Pb effects in the *in vitro* studies (Figure 5). In contrast, the significant *in vivo* changes in serum levels of sclerostin in response to Pb, more likely generated by osteocytes, provide a stronger argument for the role of this Wnt inhibitor in contributing to the Pb effects.

NEFA also depressed β-catenin expression and activity *in vitro,* but HFD caused no apparent increase in Wnt inhibitors. Thus the mechanism by which HFD diminishes β-catenin activity and promotes a low bone mass phenotype (Baron and Kneissel 2013) is distinct from that of Pb and may be more distal in the Wnt signaling pathway. Nonetheless, it can be concluded that both Pb and HFD inhibit Wnt signaling and together lead to less bone mass than either alone.

Lrp5 and the Wnt/β-catenin signaling pathway play a critical role in bone development and homeostasis. The exact mechanism by which Lrp5 signaling controls MSC fate determinations, mechanical loading, osteoblast maturation, and proliferation is still under investigation. It is quite feasible that inhibiting the Wnt pathway at this proximal step (i.e., antagonism of LRP5/6), as Pb appears to do, may lead to different outcomes than when Wnt signaling is inhibited at a more distal site (i.e., β-catenin–mediated transcription), as the HFD appears to do. This could possibly explain the disparity between Pb and the HFD on bone phenotype. For instance, sclerostin is also known to antagonize bone morphogenic protein signaling by interfering with bone morphogenetic protein molecule secretion (Krause et al. 2010), which could help explain the pronounced deficit in osteoblast activity following Pb exposure.

Effects of increased body weight and hyperlipidemia on bone tissue are complex. The conventional dogma argues that in adults of normal weight, increased mechanical force and load bearing results in greater bone mass and strength (Reid 2010). However, recent studies involving obesity suggest an inverse relationship between percent fat mass and bone mass (Dimitri et al. 2010; Fu et al. 2011; Kawai and Rosen 2010). In the present study, HFD in mice caused changes in the bone marrow environment resulting in elevated adipocyte numbers and elevated PPAR-γ activity. An increase in bone marrow adiposity is associated with osteoporotic bone loss under some conditions (Kawai et al. 2012). In addition, a recent report by Dimitri et al. (2010) showed an inverse relationship between body adiposity and bone mass in pediatric obesity, with adiposity associated with a higher proportion of bone fractures.

Several nutritional states, including obesity, produce a pro-inflammatory/pro-oxidative state in bone that appears to inhibit bone formation and increases resorption via the RANK-RANKL signaling pathway (Chen et al. 2010a). Pb also has been shown to cause an inflammatory state and can redox cycle to produce reactive oxygen species (ROS) (Cheng and Liu 2005). Thus, inflammation and ROS from Pb and/or HFD could contribute to the observed low-bone-mass phenotypes reported here.

An HFD clearly leads to an obese phenotype in C57BL/6J mice. Pb may also act as an obesogen. Elevated fasting glucose levels, increased serum leptin, and increased PPAR-γ signaling, as reported here, are evidence of this. Low-level Pb has also been indicated to increase insulin resistance (Palacios et al. 2012). The effect of Pb plus HFD on the diabetic phenotype requires additional study considering the high risk for exposure to both of these environmental factors.

Although sharing many common characteristics, Pb and HFD caused bone alterations that were distinct. Nonetheless, together they worsened the bone phenotype by potentially disrupting different aspects of Wnt signaling. The consequence of cumulative low-level Pb and obesity raises concern for these comorbid risk factors for osteoporosis. Further studies could explore the potential to remediate the HFD- and Pb-induced pathologies by recovering Wnt signaling. Furthermore, these findings argue for the need to generate more dynamic models of environmental exposures in order to establish cumulative risk for levels of Pb in the presence of co-occurring toxicants.

## Supplemental Material

(132 KB) PDF
