# Supplementary material for: Effects of Combined Exposure to Lead and High-Fat Diet on Bone Quality in Juvenile Male Mice
Source: Environ Health Perspect. 2015 Apr 10;123(10):935–43. doi: 10.1289/ehp.1408581 (PMC4590747; doi:10.1289/ehp.1408581)
Supplement: (132 KB) PDF [file ehp.1408581.s001.acco.pdf]

Environ Health Perspect
doi: 10.1289/ehp.1408581

Note to Readers: *EHP* strives to ensure that all journal content is accessible to all readers. However, some figures and Supplemental Material published in *EHP* articles may not conform to 508 standards due to the complexity of the information being presented. If you need assistance accessing journal content, please contact ehp508@niehs.nih.gov. Our staff will work with you to assess and meet your accessibility needs within 3 working days.

Supplemental Material

Effects of Combined Exposure to Lead and High-Fat Diet on Bone Quality in Juvenile Male Mice

Eric E. Beier, Jason A. Inzana, Tzong-Jen Sheu, Lei Shu, J. Edward Puzas, and Robert A. Mooney

Table of Contents

Materials and Methods

Figure S1. Pb and diet exposure timeline.

Materials and Methods

Animals: 21 timed pregnant C57BL/6J female mice were obtained from Jackson Laboratory on day 7 of gestation. The 21 litters yielded 150 pups, 78 males and 56 females. The average litter size was 6.4, with the lowest being 4 pups and the highest being 8 pups. Supplemental figure 1 denotes male mice allocation by time point. Animals were housed using micro-isolator technology with corn cob bedding, 1 litter per cage. Animals were kept on a 12hr light/dark cycle at room temperature (70-74°F, humidity 40-45%). Cages were changed on a weekly schedule. On day 21, animals were weaned and placed in cages with 3-5 mice/cage.

Glucose tolerance testing was performed on LFD (n = 5), HFD-fed (n = 5), Pb+LFD (n = 5), and Pb+HFD (n = 5) mice at 11 weeks on diet. Mice were fasted for 24 hr, anesthetized with isoflurane (5%) and tail vein blood was sampled using a commercially available glucometer (One Touch Ultra; Lifescan, Inc.). A glucose bolus (300 mg/kg) was then injected intraperitoneally. Additional glucose levels were obtained at 15, 30, 60, 100, and 150 minutes, with isoflurane again employed to ensure anesthetic plane for each blood draw. To quantify metabolic status, the net area under the curve (AUC) was calculated from the GTT curve of each mouse.

Animals were harvested late morning in a surgical suite within the laboratory. Mice were weighed on a tabletop scale, then were injected intraperitoneally with a mixture of 100 mg/kg ketamine and 10 mg/kg xylazine. Mice underwent cardiac exsanguination to collect blood, and then bone tissues were collected. After collecting whole blood, 50 μL were pipetted to a testing vial provided by Magellan Diagnostics for measurement of Pb blood levels. Samples were collected from4 animals/group at 5, 8, 11 and 17 weeks of age. In addition, every mouse was

tested for Pb blood levels at 5 weeks of age by submandibular bleeding and collection of whole blood. Data from 5 and 17 weeks are presented in the Results section. Pb blood levels in untreated mice were below the limit of detection. Samples were stored at 4°C for up to 1 week before testing. Remaining whole blood was allowed to clot for 20 min at RT, then centrifuged 30 min at 4°C at 14000 rpm. Serum supernatants were stored at -80°C until ELISA measurements were conducted.

Distilled water was used as control water, with Pb levels that were below the limit of detection by atomic absorption. Pb acetate was added to make a 50 ppm solution.

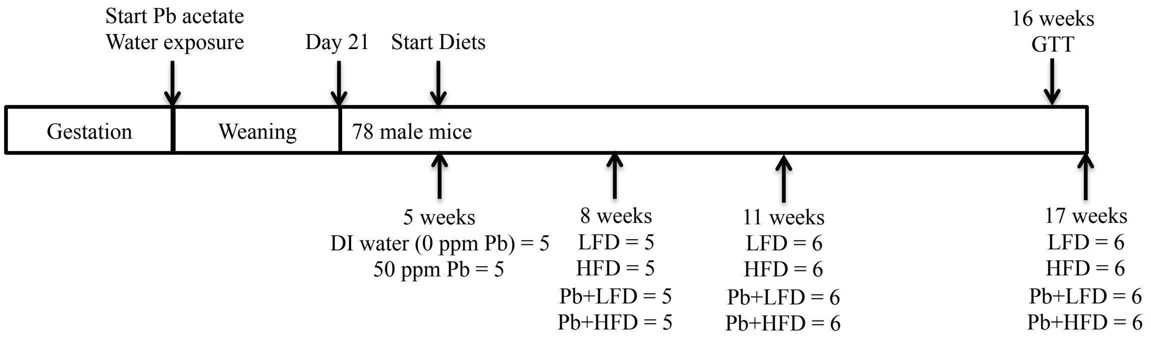

Figure S1. Pb and diet exposure timeline.
